# Reproducibility Examination of Histopathological Growth Patterns of Liver Metastases in a Retrospective, Consecutive, Single-Center, Cohort Study with Literature Review

**DOI:** 10.3390/medsci13040220

**Published:** 2025-10-03

**Authors:** Anita Sejben, Szintia Almási, Boglárka Pósfai, Bence Baráth, Ádám Ferenczi, Parsa Abbasi, Tamás Zombori, Tamás Lantos

**Affiliations:** 1 Department of Pathology, Albert Szent-Györgyi Medical School, University of Szeged, Állomás utca 1, 6725 Szeged, Hungary; 2 Department of Medical Physics and Informatics, Albert Szent-Györgyi Medical School, University of Szeged, 6720 Szeged, Hungary

**Keywords:** liver metastases, histopathological growth patterns, intraclass correlation, Fleiss’ kappa, reproducibility

## Abstract

Objectives: Histopathological growth patterns (HGPs) of liver metastases have been shown to possess prognostic significance. To date, only 2 studies have evaluated the reproducibility of HGP assessment. The aim of our study was to assess the interobserver reproducibility of HGP classification in liver metastases. Methods: A retrospective, consecutive, single-center cohort study was conducted, including patients who underwent surgical resection for liver metastases at the University of Szeged between 2011 and 2023. A comprehensive database was established, incorporating basic histopathological data for each case. Histological slides were independently reviewed by 2 pathologists, 3 pathology specialist trainees, and 2 medical students with varying levels of experience in gastrointestinal pathology. Interobserver agreement was evaluated using intraclass correlation coefficients (ICC) and Fleiss’ kappa. Results: The study included resection specimens from 205 patients, comprising 336 metastatic lesions, predominantly of gastrointestinal origin (*n* = 188). Excellent interobserver agreement was observed among specialist trainees (ICC = 0.911) and board-certified pathologists (ICC = 0.984). Overall agreement among all 7 evaluators was good (ICC = 0.822). Conclusions: Our findings demonstrate that HGPs can be reliably assessed by individuals with at least 2 years of experience in general pathology. To our knowledge, this is the first study to include the largest number of board-certified pathologists and pathology specialist trainees in a HGP reproducibility analysis. Additionally, no comprehensive literature review on this topic has been previously conducted.

## 1. Introduction

Disseminated tumours remain the leading cause of cancer-related mortality worldwide. Owing to the liver’s anatomical location and dual blood supply, liver metastases are common, particularly in association with gastrointestinal and pulmonary malignancies. As a result, they represent a major focus of interest in both pathology and oncology. Approximately one-fourth of patients with colorectal carcinoma present with liver metastases at the time of diagnosis, and an additional 25% are expected to develop hepatic metastases during the course of the disease [1,2].

Histopathological growth patterns (HGPs) were defined for hepatocellular carcinomas by Nakashima et al. in 1982, and secondary adenocarcinomas by Terayama et al. in 1996 [3,4]. HGPs are primarily used to assess prognosis following surgical resection and have been most extensively studied in colorectal adenocarcinomas. Several retrospective, consecutive studies have also validated their diagnostic utility in broader clinical settings [2,5,6]. However, it is important to emphasize that due to the biological heterogeneity of tumours and variations in treatment protocols, the utility of HGPs in a consecutive clinical setting remains insufficiently understood and not widely accepted. One of the key advantages of HGP assessment is that it can be performed using standard light microscopy. Although 5 distinct subtypes have been described, 3 of them, including replacement, desmoplastic and pushing patterns account for the vast majority of cases, allowing for practical application as a three-tiered classification system. Morphologically, the replacement pattern shows continuity with the tumour cells, while the desmoplastic pattern represents a wide desmoplastic rim that serves as a barrier between the tumour cells and hepatocytes. The pushing pattern can be characterized by a solid growth of tumour cells. In case of sinusoidal pattern, cancer cells infiltrate either in the sinusoidal vessels, or the Disse spaces. The portal pattern exhibits tumorous infiltration within the portal tracts, and/or biliary branches.

Prognostic outcomes appear to follow a descending order across growth patterns, including desmoplastic, pushing, and replacement HGPs. Owing to the rarity of sinusoidal and portal HGPs, reliable prognostic data for these subtypes are lacking; however, Latacz et al. recommend their distinction from other categories [7]. Several studies have underscored the need for a simplified classification system restricted to desmoplastic versus non-desmoplastic cases, while evidence consistently indicates that tumors exhibiting a purely desmoplastic pattern are associated with more favorable outcomes, whereas even a minor admixture of other patterns is linked to a 37–78% reduction in 5-year overall survival [1,7]. In alternative approaches, a dominant growth pattern has been identified during evaluation, with the replacement pattern correlating with poorer prognosis. It is noteworthy, however, that such cut-off definitions have thus far been established exclusively in colorectal carcinoma metastases, and these thresholds may not be directly applicable to other tumor entities [7].

Although approximately 500 publications currently address HGPs, only 2 studies to date have specifically investigated the reproducibility of HGP assessment. As a result, validation remains incomplete, and HGPs have yet to influence therapeutic decision-making in clinical practice [1,8].

Furthermore, it has to be emphasized that the concept of HGPs has not yet been universally accepted and is not currently integrated into clinical decision-making. For this reason, in our work, we evaluated all established subtypes—replacement, desmoplastic, pushing, portal, and sinusoidal—in a consecutive setting for the first time in the literature rather than adopting the simplified dichotomy of desmoplastic versus non-desmoplastic patterns. Our objective was to assess the originally defined HGPs in order to more accurately determine their reproducibility. It should also be noted that the two-tiered system was originally proposed for colorectal carcinoma metastases and has not yet gained full acceptance for other histological tumor types. It has been hypothesized that distinct cut-offs should be defined for each tumor subtype; however, no concrete steps were taken. Our paper is the first to provide a literature review on reproducibility of HGPs, as well.

## 2. Materials and Methods

### 2.1. Patient Selection and Eligibility Criteria

This retrospective, consecutive, single-center, cohort study included patients with secondary liver malignancies treated at the University of Szeged between 2011 and 2023. Patient identification was based on the International Classification of Diseases (ICD) code C78.70 (Secondary malignant neoplasm of liver and intrahepatic bile duct). Only patients who underwent surgical resection and had available pathological specimens were included. All surgeries were performed at the Department of Surgery, University of Szeged.

Clinical data were extracted from medical records, including patient age, gender, histological subtype of the primary tumour, date of diagnosis, largest macroscopic tumour diameter, TNM classification, clinical stage, and treatment received for the primary tumour. Regarding the liver metastases, the following information was collected: date of diagnosis, intrahepatic localization, type of surgical procedure, largest macroscopic diameter, completeness of resection, and presence of vascular invasion. Additional data included the presence of further metastases (if applicable) and the date of last follow-up.

Recurrence-free survival (RFS) was defined as the interval between the diagnosis of liver metastasis and either disease recurrence or patient death. Overall survival (OS) was defined as the time from diagnosis of the primary tumour to death. Time to recurrence (TTR) was defined as the interval between the diagnosis of liver metastasis and the detection of a subsequent (tertiary) metastatic lesion. Upon completion of data collection, all patient-identifiable information was removed to ensure confidentiality.

### 2.2. Evaluation of HGPs

**Figure 1:**
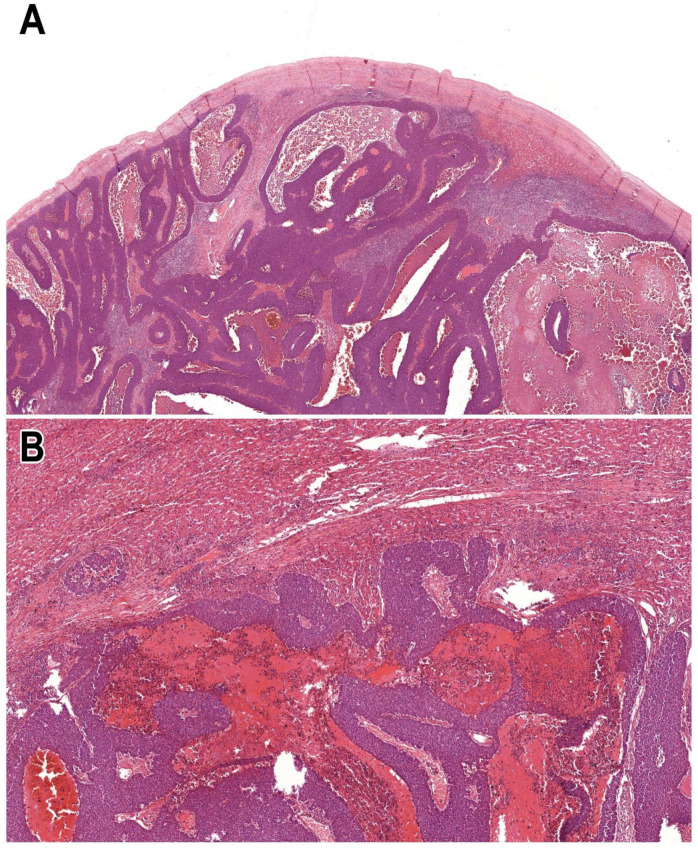
Microscopic features of subcapsular metastasis of gallbladder neuroendocrine tumour, resulting in ‘pseudo-desmoplastic’ pattern ((**A**); HE, 2×), while the rest of the tumour reflected pushing pattern ((**B**); HE, 5×).

Hematoxylin and eosin (H&E)-stained histological slides were retrieved from the archives of the Department of Pathology. Slides containing only tumour tissue without adjacent tumour-free liver parenchyma were excluded. Additionally, subcapsular metastases were omitted, as these lesions may exhibit a ‘pseudo-desmoplastic’ pattern that could confound accurate HGP assessment (Figure 1). All metastatic foci within each case were evaluated individually.

A total of 7 evaluators, each with varying levels of experience in gastrointestinal pathology and no prior exposure to HGP assessment, participated in the study. The group included 2 board-certified pathologists (AS, TZ), each with a minimum of 2 years of diagnostic experience; 3 pathology specialist trainees (BB, SA, BP), also with at least 2 years of pathology training; and 2 medical students (ÁF, PA), both of whom had completed one year of pathology coursework and had been involved in departmental scientific research activities for at least a year.

All evaluations were performed in accordance with the criteria outlined by Latacz et al. [7]. Prior to assessment, all evaluators completed a brief training session on HGP classification, during which a presentation was introduced to all participants, regarding the concept of HGPs, with their current clinicopathological significance, emphasizing the morphological criteria set by Latacz et al. [7]. At the end of the training process, an example for each subtype was presented to the evaluators, and a consensus was made. Each evaluator independently reviewed all H&E slides using light microscopy (Olympus BX53F), without collaboration or discussion with other participants. Due to the fact that there currently are no established cut-offs for tumours for non-colorectal histological subtypes, in our study, for each metastatic focus, a single dominant HGP, being present in more than 51% of a metastatic focus was assigned based on the semi-quantitative proportion of its presence. Additional tissue sections (deeper cuts) were prepared if needed to ensure accurate evaluation.

### 2.3. Statistical Analysis

Statistical analysis was carried out by SPSS Statistics V.22.0 software (IBM, SSPS 22.0, Armonk, NY, USA). To determine reproducibility, intraclass correlation (ICC; two-way random effects, absolute agreement, single rater) and Fleiss’ kappa were used. The ICC results were interpreted according to the guidelines of Koo and Li, while the kappa values were interpreted according to Landis and Koch [9,10,11].

## 3. Results

### 3.1. General Patient and Clinicopathological Data

This retrospective, consecutive, single-center cohort study included resection specimens from 205 patients, encompassing a total of 336 metastatic liver foci. The male-to-female ratio was 116:89. The mean patient age was 68 years (median: 69.5; range: 27–93 years). The average maximum diameter of the primary tumours was 34.6 mm (median: 31 mm), closely comparable to that of the liver metastases (mean: 34 mm; median: 29 mm). In 114 cases, liver metastases were unifocal (median: 1 focus; range: 1–7).

Only cases in which there was a minimum interval of 6 months between the diagnosis of the primary tumour and the detection of liver metastases were included. The majority of cases were of colorectal origin (n = 163), with a total of 188 cases classified as gastrointestinal in origin. A detailed summary of all histological subtypes is provided in Table 1.

In terms of clinical staging, most primary tumours were stage III (n = 85) or stage II (n = 46), and the majority were histologically graded as moderately differentiated (grade 2; n = 162). A total of 128 patients received adjuvant chemotherapy, while 36 patients received neoadjuvant therapy, as well, including 33 patients with colorectal carcinoma, and a gastric, a gallbladder, and an NST carcinoma case. Altogether 17 patients were given combined chemotherapy and irradiation, in 14 cases solely chemotherapy was applied, and in 2 cases, combined chemo- and immunotherapy was initiated.

The mean RFS was 31.5 months (range: 1–131 months), mean OS was 19.4 months (range: 6–58 months), and mean TTR was 18 months (range: 1–81 months).

### 3.2. Reproducibility of HGPs

**Figure 2:**
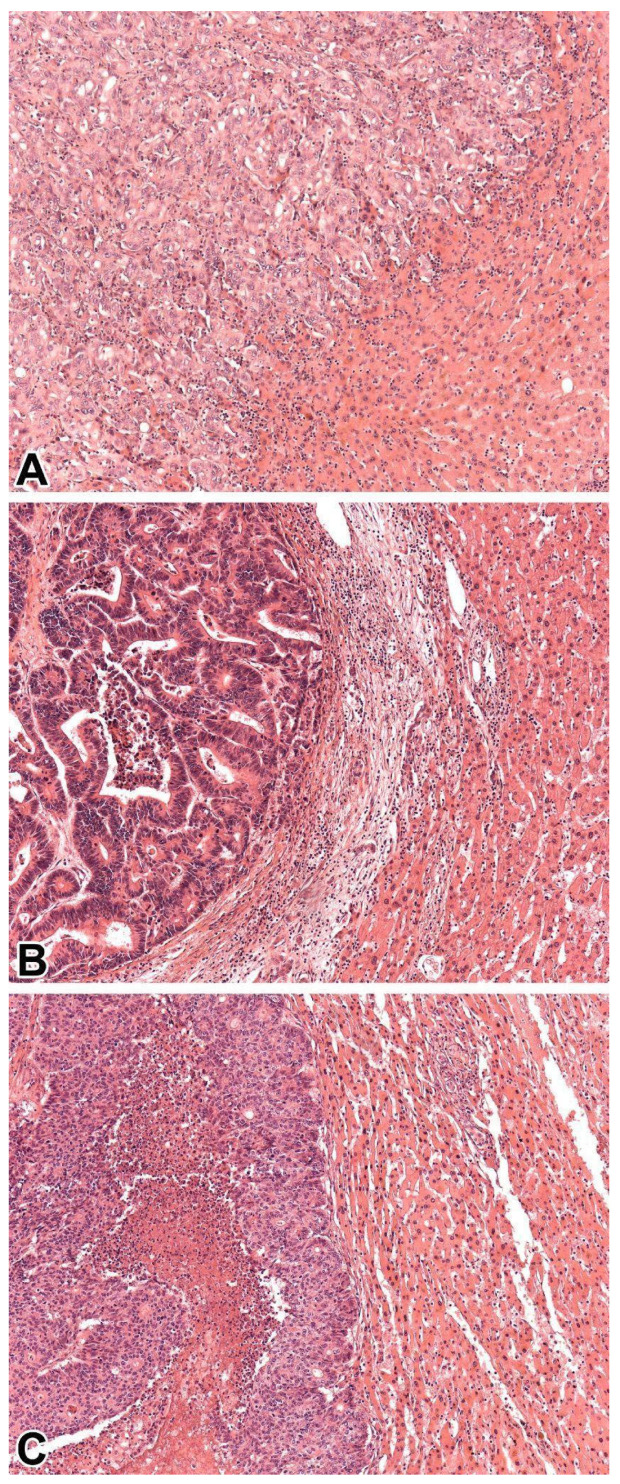
Examples for the microscopic appearance of the histopathological growth patterns observed in the study, such as replacement (**A**): Pancreatic ductal adenocarcinoma metastasis (HE, 10×), desmoplastic (**B**): Conventional colon adenocarcinoma metastasis (HE, 10×) and pushing (**C**): Conventional colon adenocarcinoma metastasis (HE, 10×).

Our cohort did not include cases with dominantly sinusoidal or portal patterns. Figure 2 illustrates replacement, desmoplastic, and pushing patterns found in our study. The most prevalent pattern proved to be replacement type, followed by desmoplastic, and pushing pattern. These patterns are displayed in order of occurrence on Figure 2. Evidently, pushing pattern was exceedingly commonly observed in NETs and NECs [12]. Moderate agreement (ICC score: 0.567) was found between the medical students, while excellent agreement was reached between both the pathology specialist trainees (ICC score: 0.911) and the board-certified pathologists (ICC score: 0.984). The general agreement between all 7 evaluators proved to be good, with an ICC score of 0.822. Table 2 summarizes the results of ICC correlation.

Moderate agreement (kappa = 0.532) was reached by the medical students, and excellent agreement was found both between the specialist trainees (kappa = 0.897) and the pathologists (kappa = 0.971). The overall agreement proved to be substantial (kappa = 0.793). Table 3 highlights the Fleiss’ kappa results.

## 4. Discussion with Literature Review

The reproducibility of the assessment of HGPs has not yet been widely examined. For the literature search, the keywords “histopathological growth pattern”, “reproducibility”, “reliability”, and “replicability” were used in PubMed between 1980 and 2024. The search was carried out on 31^st^ March 2025. All studies with this aim have been included, and currently, only 2 publications are available regarding the matter.

Following the establishment of standardized guidelines by the Liver Metastasis Research Network, van Dam et al. conducted a study involving 374 patients with liver metastases from colorectal and breast carcinomas. In each case, a predominant HGP was identified, defined as comprising at least 50% of the tumour–liver interface. For the reproducibility assessment, 12 evaluators—of whom only 4 had prior experience with HGP scoring, and 3 were board-certified pathologists—were included after a standardized training session. Interobserver agreement was assessed using intraclass correlation coefficients (ICCs). However, the thresholds for interpretation were determined independently by the authors, with ICC values > 0.05 considered “good” and >0.07 considered “excellent,” which deviates from widely accepted statistical conventions. Although the abstract reports good-to-excellent agreement for the classification of desmoplastic and replacement-type HGPs, specific ICC values are not provided in the main text. Instead, a heat map is used to illustrate interobserver concordance. The authors noted that evaluators were confident in distinguishing typical desmoplastic and replacement-type 1 patterns. Importantly, the study concluded that, according to their proposed criteria, HGP assessment is reproducible and that desmoplastic HGPs are significantly associated with improved OS compared to replacement and pushing subtypes (*p* = 0.006) [1].

The second study assessing the reproducibility of HGP evaluation was published in 2019 by Höppener et al. Following 2 structured training sessions, a total of 363 colorectal carcinoma liver metastases were assessed by a board-certified pathologist and a PhD student, both of whom had no prior experience in HGP evaluation. HGPs were assessed both within and between metastatic foci. Cohen’s kappa coefficient was employed to evaluate intra- and interobserver reproducibility. Intraobserver agreement was assessed after the first and second training sessions. For the pathologist, the kappa values were 0.836 and 0.953, respectively, indicating excellent reproducibility. Similarly, the PhD student achieved kappa values of 0.747 and 0.951, reflecting substantial to excellent agreement. Interobserver variability was notably different after the first training session, with the pathologist and PhD student achieving kappa values of 0.836 and 0.747, respectively. However, in the second round, their performance was nearly identical, with kappa values of 0.953 and 0.951, demonstrating excellent interobserver agreement following training [8]. Table 4 summarises all the currently available publications, incorporating the results of our study.

In this retrospective cohort study, we aimed to evaluate the reproducibility of HGP classification using 7 independent evaluators with diverse educational backgrounds: 2 board-certified pathologists, 3 pathology specialist trainees, and 2 medical students. The study included patients diagnosed with secondary liver malignancies who underwent surgical treatment at the University of Szeged between 2011 and 2023. Patient selection was based on ICD code C78.70 (secondary malignant neoplasm of liver and intrahepatic bile duct), and only cases with available surgical resection specimens were included.

H&E-stained slides were retrieved from the archives. Cases containing only tumour tissue without adjacent tumour-free liver parenchyma, as well as subcapsular metastases were excluded. All evaluators independently assessed each metastatic focus. A single dominant HGP was assigned per lesion, based on the semi-quantitative estimation of the most prevalent pattern at the tumour–liver interface.

During the evaluation process, the evaluators faced challenges. According to their subjective perception, those cases with primary and secondary tumour diagnosis at the same time, combined with neoadjuvant therapy of the primary tumour were problematic, while neoadjuvant therapy naturally altered the histological appearance of the metastases, as well, inducing necrosis or the tumour with consequential abnormal fibrotic tumour bed formation, named as ‘pseudo-desmoplastic pattern’ [13].

Excellent agreement was reached between both the pathology specialist trainees (ICC score: 0.911) and the board-certified pathologists (ICC score: 0.984), while moderate agreement was seen between the medical students (ICC: 0.567). The general agreement between all 7 evaluators proved to be moderate, with an ICC score of 0.822. Regarding Fleiss’s kappa, excellent agreement was found both between the specialist trainees (kappa = 0.897) and the pathologists (kappa = 0.971), while moderate agreement (kappa = 0.532) was reached by the medical students. The overall agreement proved to be substantial (kappa = 0.793). In spite of the promising results, it has to be emphasized that these results solely reflect the experience of a single institution and may not be generalizable.

A strength of this work is its novelty. Previous research has primarily focused on the prognostic implications of HGPs, but the reproducibility of their classification has remained largely unaddressed. To our knowledge, this is the first reproducibility study that incorporates the largest number of board-certified pathologists, pathology specialist-trainees, and medical students, to date, and no comprehensive literature review on this aspect of HGPs has yet been conducted. By systematically examining interobserver variability across evaluators with different levels of expertise, our study fills an important gap in the field. Even though there is a paradigm shift in the evaluation process of HGPs, regarding evaluating them based solely on the presence of desmoplastic pattern, the concept of HGPs itself is not completely accepted and it is not yet integrated into any decision-making process; therefore, the authors chose to evaluate all currently accepted subtypes, including replacement, desmoplastic, pushing, portal, and sinusoidal. Our aim was to determine the originally defined HGPs, instead of adapting to the new, easier concept, consequently, our results reflect the accurate reproducibility of HGPs. Moreover, we identified specific challenges in interpretation, such as alterations induced by neoadjuvant therapy, which may lead to pseudo-desmoplastic appearances. These observations underscore the need for standardized evaluation criteria and highlight the potential value of developing consensus guidelines and training programs. Although the findings are based on a single-institution cohort and therefore require validation in larger, multi-center studies, even international studies, they provide a critical first step toward establishing HGP classification as a reproducible, clinically applicable tool. To enhance comparability and reliability, future evaluation studies should consider applying a standardized approach. First, it remains to be determined whether the two-tiered system (desmoplastic vs. non-desmoplastic) or the five-tiered system (replacement, desmoplastic, pushing, sinusoidal, and portal) more accurately reflects prognosis and shows stronger associations with clinicopathological factors. This decision will have important implications for the design of future evaluation processes. Our study was conducted using the five-tiered system, with the cut-off value of 51% for the dominant pattern defined by the authors, while cut-off values have not yet been established for non-colorectal carcinoma cases. Accordingly, our approach—comprising a short training session and a standardized evaluation method—is specifically tailored to the original HGP framework and can be reproduced by other researchers pursuing the same objectives. By demonstrating both the feasibility and limitations of current practice, this study sets the stage for broader adoption and standardization of HGP assessment in routine diagnostic pathology.

## Figures and Tables

**Table 1:** Summary of the examined tumours’ histological subtype.

Primary Tumour Histological Subtype	Number of Cases
Colonic adenocarcinoma NOS	102
Colonic mucinous adenocarcinoma	5
Colonic leiomyosarcoma	1
Rectal adenocarcinoma NOS	52
Rectal mucinous adenocarcinoma	4
Gastric intestinal adenocarcinoma	4
Gastric NET	1
Small intestinal NET	3
Small intestinal NEC	2
Pancreatic ductal adenocarcinoma	3
Pancreatic NET	2
Gallbladder carcinoma	4
Extrahepatic bile duct carcinoma	4
Gallbladder NET	1
NST carcinoma	4
Pulmonary small cell lung carcinoma	1
Pulmonary basaloid squamous cell carcinoma	1
Nasopharyngeal squamous cell carcinoma	1
Hypopharyngeal squamous cell carcinoma	1
Prostatic adenocarcinoma	1
Papillary renal cell carcinoma	1
Urothelial carcinoma	1
Cervical squamous cell carcinoma	1
TFE3-rearranged renal cell carcinoma	1
Mixed germ cell tumour *	1
Malignant melanoma	1

NEC—Neuroendocrine carcinoma, NET—Neuroendocrine tumour, NOS—Not otherwise specified, NST—No special type carcinoma, TFE3—Transcription factor E3. *: 50% yolk sack tumour, 50% postpubertal teratoma.

**Table 2:** Results of intraclass correlation (ICC), based on the evaluators’ training background (CI—confidence interval).

Evaluator	ICC Score	CI (95%)	ICC Scale	Interpretation [9]
**Pathologist**	0.984	0.980–0.987	<0.5	Poor
**Specialist trainee**	0.911	0.894–0.926	95%CI < 0.5	Moderate to poor
**Medical student**	0.567	0.490–0.636	0.5–0.749	Moderate
**Altogether**	0.822	0.796–0.847	95%CI > 0.749	Good to moderate
			0.75–0.9	Good
			>0.9	Excellent

**Table 3:** Results of Fleiss’ kappa for reliability of agreement, based on the evaluators’ training background (CI—confidence interval).

Evaluator	Fleiss’ Kappa	CI (95%)	Kappa Scale	Interpretation [11]
**Pathologist**	0.971	0.969–0.974	−1 to −0.01	Poor
**Specialist trainee**	0.897	0.896–0.899	0–0.10	Slight
**Medical student**	0.532	0.529–0.534	0.11–0.20	Slight
**Altogether**	0.793	0.792–0.793	0.21–0.30	Fair
			0.31–0.40	Fair
			0.41–0.50	Moderate
			0.51–0.60	Moderate
			0.61–0.70	Substantial
			0.71–0.80	Substantial
			0.80–0.90	Almost perfect
			0.9–1	Almost perfect

**Table 4:** Summary of literature review regarding reproducibility studies on histopathological growth patterns of liver metastases (ICC—Intraclass correlation).

Author and Year of Publication	Case Number (n)	Number of Evaluators (n)	Educational Background	Identifiable Patterns	Statistical Method	Training Session	Results
van Dam et al., 2017 [1]	152	12	3 pathologists, 9 scientists	Replacement, desmoplastic, pushing, sinusoidal, portal	ICC	Teaching and training set	Good-to-excellent agreement (ICC > 0.5) between evaluators, regardless of educational background
Höppener et al., 2019 [8]	825 within, 363 between	2	1 pathologist, 1 PhD student	Replacement, desmoplastic, pushing	Cohen	2 training sessions	Within k = 0.953; between k = 0.0951
Our study, 2024	205 cases with 336 foci	7	2 pathologists, 3 pathology specialist trainees, 2 medical students	Replacement, desmoplastic, pushing, sinusoidal, portal	ICC, Fleiss’ kappa	Short training session	Excellent agreement between pathologists and pathology specialist trainees (ICC: 0.984 and 0.911). Moderate agreement between medical students (ICC: 0.567). Good general agreement between all evaluators (ICC: 0.822). Excellent agreement between pathologists and pathology specialist trainees (kappa = 0.971 and kappa = 0.897). Moderate agreement between medical students (kappa = 0.532). Substantial overall agreement (kappa = 0.793).

## Data Availability

The data that support the findings of this study are not openly available due to reasons of sensitivity and are available from the corresponding author upon reasonable request.

## Abbreviations

The following abbreviations are used in this manuscript:

CI	Confidence interval
HE	Hematoxylin and eosin
HGP	Histopathological growth pattern
ICC	Intraclass correlation
ICD	International Classification of Diseases
NEC	Neuroendocrine carcinoma
NET	Neuroendocrine tumour
NOS	Not otherwise specified
NST	No special type carcinoma
OS	Overall survival
RFS	Recurrence-free survival
TFE3	Transcription factor E3
TTR	Time-to-recurrence
